# DECIPHERING THE CHEMICAL LANGUAGE OF GUT BACTERIA IN AGING AND LONGEVITY

**DOI:** 10.1093/geroni/igae098.1592

**Published:** 2024-12-31

**Authors:** Shuo Han

**Affiliations:** Duke University School of Medicine, Durham, North Carolina, United States

## Abstract

The human gut microbiota encodes diverse metabolic pathways, where gut microbes make numerous compounds that are relevant to human health and hold untapped therapeutic potential. Fecal microbiota transplantation has been shown to delay age-associated decline in mouse and fish models. However, how specific gut bacteria and their metabolites impact host physiology represent a new frontier that remains to be fully explored. Leveraging our expertise in the gut microbiome and aging biology, we tackle this underexplored area from both the microbial and host’s perspectives. Specifically, our lab seeks to 1) mechanistically characterize gut bacteria and bioactive small molecules in host aging and physiology, and 2) identify gut microbial-dependent, host cellular mechanisms underlying organismal health-span and lifespan. We aim to understand how gut bacteria mechanistically contribute to host aging and longevity and to identify new molecular targets to delay age-associated decline.

